# A Wind Tunnel Setup for Fluid-Structure Interaction Measurements Using Optical Methods

**DOI:** 10.3390/s22135014

**Published:** 2022-07-02

**Authors:** Simon Nietiedt, Tom T. B. Wester, Apostolos Langidis, Lars Kröger, Robin Rofallski, Martina Göring, Martin Kühn, Gerd Gülker, Thomas Luhmann

**Affiliations:** 1 Institute of Applied Photogrammetry and Geoinformatics (IAPG), Jade University of Applied Sciences, Ofener Str. 16/19, 26121 Oldenburg, Germany; robin.rofallski@jade-hs.de (R.R.); martina.goering@jade-hs.de (M.G.); luhmann@jade-hs.de (T.L.); 2 Institute of Physics, University of Oldenburg, ForWind, Küpkersweg 70, 26129 Oldenburg, Germany; tom.wester@uol.de (T.T.B.W.); apostolos.langidis@uol.de (A.L.); lars.kroeger@uol.de (L.K.); martin.kuehn@uol.de (M.K.); gerd.guelker@uol.de (G.G.)

**Keywords:** dynamic metrology, photogrammetry, PIV, wind tunnel, fluid-structure interaction, wind turbine

## Abstract

The design of rotor blades is based on information about aerodynamic phenomena. An important one is fluid-structure interaction (FSI) which describes the interaction between a flexible object (rotor blade) and the surrounding fluid (wind). However, the acquisition of FSI is complex, and only a few practical concepts are known. This paper presents a measurement setup to acquire real information about the FSI of rotating wind turbines in wind tunnel experiments. The setup consists of two optical measurement systems to simultaneously record fluid (PIV system) and deformation (photogrammetry system) information in one global coordinate system. Techniques to combine both systems temporally and spatially are discussed in this paper. Furthermore, the successful application is shown by several experiments. Here, different wind conditions are applied. The experiments show that the new setup can acquire high-quality area-based information about fluid and deformation.

## 1. Introduction

The interaction between a fluid and a flexible structure is a common physical phenomenon in nature. An example is the interaction of a heart valve with the surrounding blood, where different pressure distributions change the shape and position of the valve [1]. The interaction is bidirectional in other examples, such as flapping owl wings or wind turbines [2,3]. The fluid modifies the wing/blade and the deformation leads to additional turbulences. Additionally, on technical objects, fluid-structure interaction (FSI) can be observed [3,4]. Although the phenomenon occurs in many domains, its origin and impact have not yet been fully explored. Especially in technical and medical applications, the phenomenon is of great interest to open up new potential for development.

However, the investigation of FSI is very complex and strongly dependent on the specific application. Therefore, simulations are used in all domains, which are characterized by very long computation times (several days or more in some cases) for turbulent flows [3,5,6]. Furthermore, the required approximations can lead to inaccurate results. Therefore, experiments are carried out to observe FSI in reality. These investigations are very time-consuming, expensive, and not fully reproducible. Instead, depending on the application, wind tunnel tests are used, which can represent a connection between real tests and simulations. Especially by using active grids, real wind situations can be simulated [7]. However, wind tunnel tests are characterized by high complexity and high technical effort.

For example, both the inflow and the deformations or load distribution of the respective flexible object must be acquired simultaneously. Moreover, this information must be connected temporally and spatially for a comprehensive view. A possible approach is to acquire the wind flow using PIV and the surface deformations in the form of vibrations [8]. Vibrations are acquired only at a discrete point using laser interferometry. Another experiment described the acquisition of a flexible wing underwater [9]. A combination of PIV and a structured-light system is used for this purpose. Several authors used two passive optical methods to observe FSI on owl wings, mechanical wings, and wind turbines [10,11,12]. The benefit is a contactless acquisition, which reduces the manipulation of flow behavior and deformation.

The increasing size and rated capacity of the modern wind turbines, consisting of machines with blade lengths over 100 m and power output of several megawatts [13], makes FSI of great importance. FSI leads to additional loads which can damage the blades and reduce the whole turbine’s performance [14]. To further improve the turbines’ efficiency, safety, and profitability, it is crucial to consider and study this phenomenon. The knowledge gained can be considered in the development of new turbines. A well-known experiment is known as MEXICO [15]. Here, a rotating model wind turbine was used. The stereo Particle Image Velocimetry (PIV) system acquired the aerodynamic phenomena, and load measurement techniques yield the resulting loads. Numerous authors use the collected information for comparison with simulations [16,17]. Furthermore, this experiment is suitable for investigating load-reducing methods [18]. However, the setup is unsuitable for the area-based acquisition of FSI at a rotating model wind turbine.

This paper presents an experimental setup for measuring FSI on rotating wind turbines in wind tunnel tests using two optical measurement systems. Combining the two measurement techniques requires a unique synchronization unit and a series of spatial coordinate transformations. The combination allows a more comprehensive view of the FSI phenomenon and can improve engineering models. The presented experimental setup allows for non-intrusive and area-based measurement of fluid and geometry and the consideration of different influences. Furthermore, the presented experimental setup can be easily adapted for other applications. This report focuses mostly on the fusion of different data with the main emphasis on photogrammetric issues.

## 2. Measurement Techniques in Wind Tunnel Experiments

Numerous disciplines have been using wind tunnel experiments successfully for decades. The employed measurement techniques are as individual as the experiments themselves. Different physical quantities are measured to obtain information about the investigated objects’ flow behavior, loads, and geometric deformations. Therefore, the developments in flow measurement and photogrammetric deformation analysis are explained in the following.

### 2.1. Flow Measurement Techniques

The subject of flow measurement technology covers many methods to acquire and analyze fluid flows. The fluid can be either a gas or a liquid, where different methods are suitable for respective applications. Applications, where the techniques are successfully applied, are the aerospace industry [19], the automotive industry [20], and the medical field [21]. Despite the heterogeneous field of application, it is possible to split these techniques into different categories. The classification can be made to the number of determinable velocity components (2C = two velocity components) and the number of the measurement dimensions (2D = two dimensions).

Hotwire-Anemometers (1D-3C) are widely used for time-resolved flow measurements [22,23]. The hot wire is heated electronically. Due to heat transport into the surrounding fluid, the resistance of the wire changes. By measuring the resistance, the flow velocity can then be deduced. The advantages of the measurement method are the high temporal resolution and the inexpensive and straightforward handling. A disadvantage is the intrusive concept of the sensor. This interaction can manipulate the flow and the uniqueness of direction.

As an alternative, non-intrusive optical methods are suitable for investigating large flow field areas. Small tracer particles are added to the fluid to track the flow itself. Those small particles follow the flow without any slip and have a size depending on the application. Typical sizes range from 0.5 µm to 100 µm [24]. Laser Doppler Anemometry (LDA/LDV) detects the wind velocity component at a discrete location [25]. It consists of two laser beams that intersect in the measurement volume. Due to the coherent laser light, the intersection creates an interference pattern. Particles moving through the interference pattern scatter the light and produce a light signal recorded by a photomultiplier. The flow velocity component perpendicular to the interference fringes can be derived from the frequency of the recorded burst signal. In addition to non-intrusive detection, LDA is characterized by a high temporal resolution comparable to hot wire applications.

Another optical method is known as Particle Tracking Velocimetry (PTV). The measurement setup consists of at least one camera and a high-power laser. A light sheet is formed from the laser to illuminate an entire plane. As with LDA, tracers are added to the fluid. The acquisition is made by the camera optically in several time steps. Digital image processing methods then analyze the acquired image sequence. PTV aims to detect individual particles in the images and determine the trajectories with as few gaps as possible. Cross-correlation and least-squares matching (LSM)-based approaches can be used as tracking methods [26]. To fortify the method against short-term occlusions, ref. [27] extends the tracking model by a Kalman filter. If additional cameras extend the experimental setup, the movements can be tracked in three dimensions (2D-3C). The use of additional cameras requires geometric calibration of the whole camera system (interior and exterior orientation). A test field is usually used for calibration. The extension to a multi-media approach is also possible [28]. This allows the analysis of liquid fluids through separate windows. In addition to the complex experimental setup, a disadvantage of the method is the number of tracers in the fluid. Only a few particles should be added to the flow to track the particles as long as possible. Furthermore, local flow behavior may not be visualized correctly.

To obtain local flow information, the particle density (seeding) can be increased, and Particle Image Velocimetry (PIV) can be used. PIV is based on the same experimental setup as PTV. However, individual particles are not tracked in the image sequence. Instead, the image is divided into smaller so-called interrogation windows (IW). Cross-correlation is usually used to extract the movement of the particles inside each IW. New approaches also include optical flow methods or deep learning techniques in the image sequence [29]. Consequently, information about a dense flow field can be obtained. PIV can be considered the standard technique in different disciplines. Especially with the development of pulsed lasers and digital high-speed cameras, the frame rate could significantly increase. For example, [30] uses a high-speed stereo PIV system (2D-3C) to analyze dynamic stalls on rotor blades. The use of high-speed cameras allows a frame rate of several kilohertz, so the approach is referred to as time-resolved PIV in literature [31]. An extension of the method by adding the third dimension to a 3D-3C method is possible. For this, ref. [32] changed the position of the laser plane after the acquisition of an image pair. This procedure creates a volume that cannot be analyzed at a discrete time. Instead of one moving laser plane, two laser planes with a calibrated baseline between each other can also be used [33]. The separation of the images is achieved by polarization. A popular technique to acquire a dense volume is tomographic PIV (TOMO-PIV) [34,35]. Here, the laser pulse illuminates a volume instead of a plane. Several cameras observe the volume from different directions. The image information can be transformed into a voxel model through digital image processing methods. Subsequently, the subscale voxels in the voxel model sequence can be tracked. For this purpose, correlation methods or LSM-based methods can be used in classical PIV [36,37]. The size of the voxel models is up to 100 cm^3^. Thus far, the approach is limited by the repetition rate and the laser’s energy [38]. In general, different aspects must be considered to apply PIV/PTV methods successfully. On the one hand, the number of tracer particles must be adjusted to the application case. Furthermore, Scheimpflug adapters are used to focus the entire FOV, and specific algorithms can be reduced the influence of so-called ghost particles. For a practical overview of the setup and the processing, see [24].

### 2.2. Optical 3D Metrology

Optical 3D measurement techniques, commonly associated with photogrammetric deformation analysis or Digital Image Correlation (DIC), can be used for non-intrusive and area-based measurement of deformations. Applications range from determining the deformation behavior of aircraft wings at discrete points to crack detection in structural loading tests [39,40].

The basic principle is the same in all applications and is closely related to PIV and PTV methods. A calibrated camera system consisting of at least two cameras is used for passive optical deformation measurement. The surface can be reconstructed in three dimensions from the synchronized image sequences for each epoch. For this purpose, semi-global matching (SGM) or LSM can be used to find correspondences between images. Subsequently, the object coordinates are determined by forward intersections. As an alternative, object space-based methods can be used, where the correspondence problem and the determination of the object point are performed in one process [41,42]. Optical flow methods can be used for tracking. A popular alternative is to use the LSM. As an example, ref. [40] uses LSM for tracking the vertices of a triangular mesh, ref. [43] for determining glacial motion, and [44] uses LSM for the reconstruction and tracking of body parts. The required approximations for temporal matching are predicted in the object domain by linear extrapolation of previous epochs projected into the image domain of the predicted epoch. Other authors also connect multiple time steps, allowing the determination of consistent spatio-temporal data without having exact prior knowledge of the motions. The connection of several time steps allows spatio-temporal loop closure for stereo matching [45]. Here, an image feature of a start image is tracked within the image sequence via a temporal matching procedure and mapped in the respective stereo partner. The image feature in the stereo partner is projected back into the original start image, where the occurring difference to the original image feature is defined as a similarity measure. Another approach is the use of a kinematic model implemented in a Kalman filter for spatio-temporal matching [46]. For a multi-view approach, ref. [47] apply a kinematic model in the form of an extended bundle adjustment. In addition to the computation of camera parameters, 3D trajectories and asynchronies can also be estimated. A two-step procedure can increase the spatio-temporal matching process [48]. In the first step, the optical flow and the reconstruction determine separately. Then, three-dimensional trajectories are determined from the results and introduced as constraints in another scene flow calculation. The procedure must be carried out twice but leads to consistent trajectories over more extended periods since no epoch is taken as a reference.

A significant drawback of passive optical methods is the required heterogeneous texture on the object surface. Areas with homogeneous textures are challenging to reconstruct by classical image matching methods. Therefore, if possible, optimizing the targeting is necessary for some applications. Area-based signalizations, characterized by high contrast, randomness, and isotropy, enable a high quality of matching [49]. A high dynamic range (16 instead of 12-bit color depth) can also lead to a better matching result [50]. An overview of the different surface targeting possibilities is given in [49]. To evaluate texture quality, different local and global criteria can be used [51]. However, the evaluation of the signalization depends on many factors and remains an open research field.

As an alternative to passive methods, active optical methods can be used. The most significant advantage of these methods is their independence from the natural texture of the object. The measurement system consists of a projector and at least one camera. The interior orientation of the camera and the relative orientation to the projector must be known to reconstruct the surface. If only one camera is used instead of two cameras, the relative orientation to the second camera must be known. During the measurement, different patterns are projected onto the object sequentially. The different patterns are acquired by the camera and then decoded. The fringes can be mapped to the different pixels from decoding, and the surface can be reconstructed. Many different variations of structured-light systems have been developed using different patterns [52]. With the development of high-speed cameras and high-power projectors, deformations can be measured with a frequency of several kilohertz [53]. For example, ref. [54] uses a structured-light system to reconstruct a wing in motion. In another example, a system reconstructs an airbag deployment at a frame rate of several kilohertz [55]. A disadvantage of the method is the limited measurement volume. Due to the projection power, the method can currently only be applied to small-scale objects.

## 3. Experimental Setup

**Figure 1:**
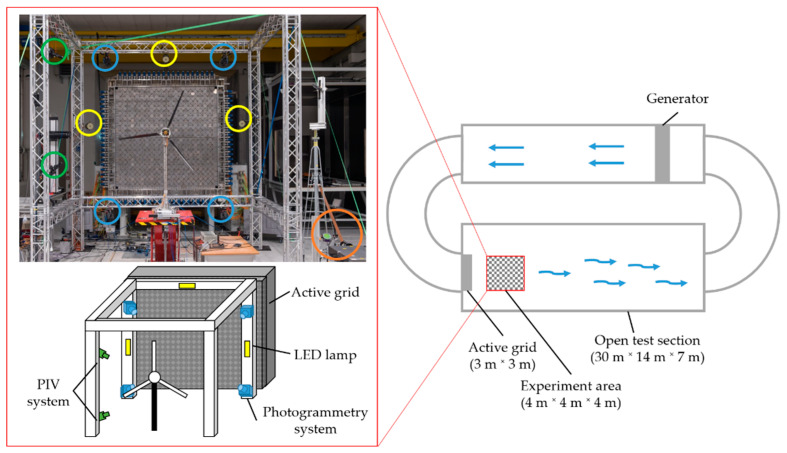
Experimental setup in the wind tunnel (**left**). Top view of the wind tunnel (**right**). PIV cameras are encircled in green, PIV laser in orange, photogrammetry in blue, LED lamps in yellow.

The experiments took place in the Turbulent Wind Tunnel of the University of Oldenburg [56,57]. The wind tunnel is built in the Göttingen design and is shown schematically in Figure 1. The test section has a length of 30 m and a cross-section of 3 m by 3 m. Experiments can take place in the closed as well as in the open test section. Due to the size of the model wind turbine, the open test section is used. For the generation of the wind flow, four 100 kW motors are installed. These generators can provide wind speeds up to 42 m/s (closed test section) or up to 32 m/s (open test section). An active grid characterizes the wind tunnel (see Figure 1), which allows the generation of realistic wind situations. It consists of 80 shafts that can be controlled individually. Each shaft is equipped with numerous diamond-shaped flaps. Thus, even complex turbulent wind situations can be simulated with high repeatability.

The left side of Figure 1 shows the test setup for detecting fluid-structure interaction. The setup consists of six high-speed cameras, four LED lamps, and a high-power laser (orange). Two high-speed cameras are used as a stereo-PIV system (green). The remaining cameras and the four LED lamps (yellow) are used for the photogrammetry system (blue).

### 3.1. Wind Turbine

**Figure 2:**
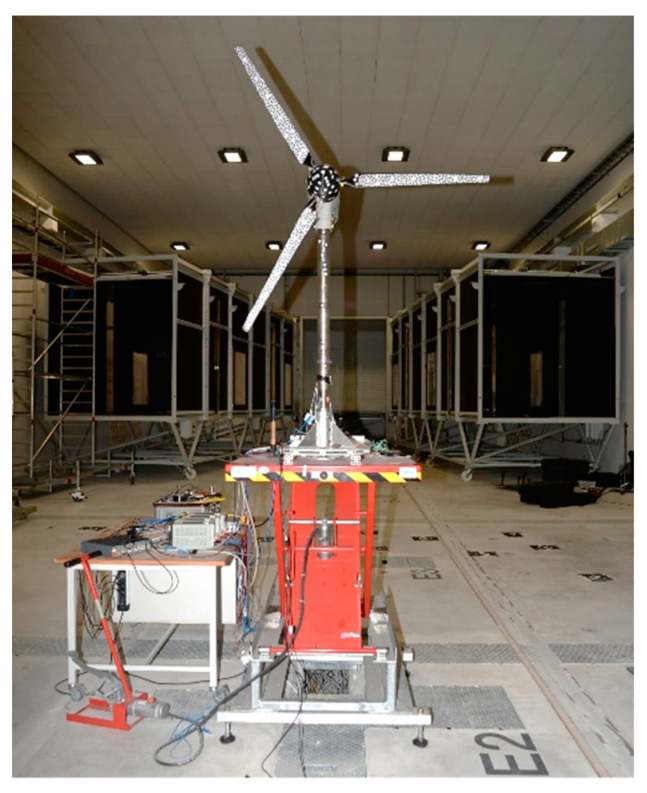
Model Wind Turbine Oldenburg (MoWiTO).

The measurements were performed on the Model Wind Turbine Oldenburg (MoWiTO) 1.8, illustrated in Figure 2. The turbine is an in-house developed model with a rotor diameter of 1.8 m. It has been designed for experiments on rotor aerodynamics and testing of control strategies. It is scaled after the NREL 5 MW reference turbine with a geometric scaling of 1:70 and a time scaling factor of 50:1. Hence, according to the geometric downscaling of the model, speed parameters must be increased accordingly.

The objective dictated the design methodology of MoWiTO 1.8 to maintain the tip speed ratio (TSR) of the reference in the wind tunnel conditions. The TSR is 7.5. The preservation of this number corresponds to the similarity of the velocity triangles on the blade profiles. Furthermore, the airfoil profiles used for the blades have similar aerodynamic behavior in the sub-scale compared to the original machine at full scale. Additional information on the wind turbine design can be found in [58]. Especially for these measurement campaigns, a new set of blades was designed and produced. These blades have a similar aerodynamic shape to the first-generation blades described above. However, they are lighter and more flexible, as demanded by the scaling relations. Each blade is constructed with carbon fiber and has a weight of 82 g [59].

The turbine also features extensive measurement and control instrumentation. Its operation is controlled through generator load regulation and pitch angle control. It is realized by an individual, independently actuated motor at the root of each blade. Loads, such as the flap-wise blade root bending and the tower fore-aft bending moments, are measured by strain gauges placed on the model. All the turbine signals for measurement and control are connected to a NI-9066 compactRio controller. Both data acquisition and turbine operation are conducted. Inside the wind tunnel, MoWiTO is being fixed on a hydraulic pump-driven platform, which brings the hub at the height of the tunnel’s centerline. The rotor is oriented perpendicular to the wind direction, at a distance of 4.7 m from the active grid. The deflection of each blade depends on the local inflow characteristics. The MoWiTO rotates approximately at 8 Hz during the experiments, which results in high tip speeds of 57 m/s. As a result of this behavior, only a camera system with a low exposure time and high frame rate (>200 Hz) can be used to measure necessary information at the tip position.

### 3.2. Particle Image Velocimetry (PIV)

A high-speed stereoscopic (2D-3C) PIV setup is used to investigate the flow around the rotor blades of the mentioned model wind turbine. The system consists of two Phantom V1212 cameras with an internal RAM of 72 GB. Each camera has a sensor size of 1280 × 800 pixels and a temporal resolution of up to 12 kHz at full-frame resolution. During the measurement campaign, a frame rate of 4 kHz was used for the recording to measure over a longer period. In addition, the sensor size was cropped to fit with the illuminated region and enable longer measurement periods. To compensate for the angle between the cameras and the subsequent measurement plane, the cameras are equipped with Scheimpflug adapters. For imaging NIKKOR lens objectives with a 200 mm fixed focal length are used. The objectives are equipped with interference filters to prevent the scattered and converted light from entering the camera.

**Figure 3:**
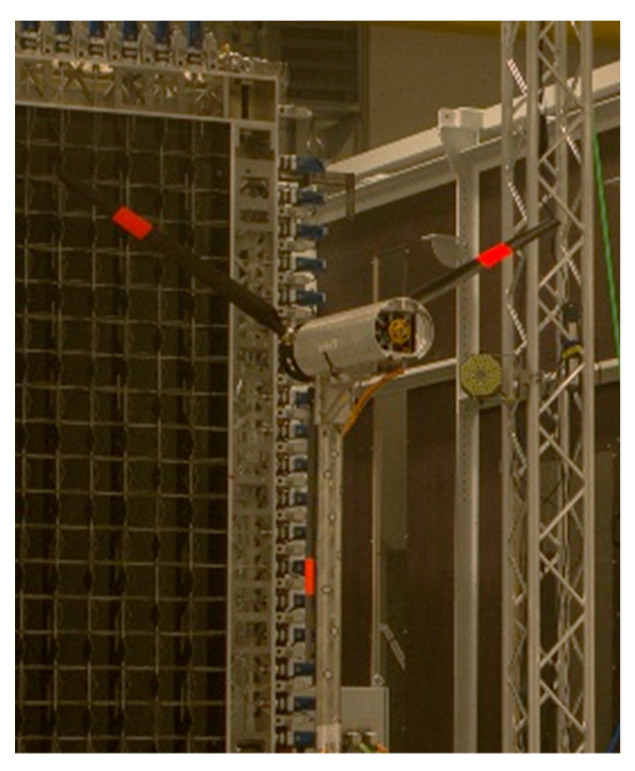
Prepared MoWiTO with red foil.

The field of view is illuminated by a dual head Nd:YAG laser of type PI DM150-532. This laser can produce pulses with energies up to 15 mJ per pulse at a repetition rate of 10 kHz. The emitted laser light is formed into a light sheet using concave and cylindrical lenses. To reduce the oversaturation of the camera images due to surface reflections, a red fluorescent foil was applied to the turbine’s blades (see Figure 3).

DEHS (Di-Ethyl-Hexyl-Sebacat) particles are used as tracers to seed the flow field. They are applied using a PIVTec PIVpart160 seeder with 160 cascadable Laskin nozzles. The mean droplet size of the seeding is 1 µm. These particles do not have an impact on the photogrammetry system.

The PIV system is calibrated using a three-dimensional calibration plate of type 204-15 from LaVision. The entire calibration, imaging, and evaluation process of the PIV measurement are done using the LaVision DaVis 8.4 software. During the evaluation, a multi-pass stereoscopic-cross-correlation with a multigrid analysis is performed with different interrogation window sizes depending on the given flow situation. The PIV measurements can achieve a spatial resolution of 0.057 mm × 0.057 mm of the velocity fields with a Field of View of 150 mm × 300 mm.

### 3.3. Photogrammetry

To obtain deformation information about the blades, a photogrammetric system is used. The high-speed camera system consists of three PCO dimax HD+ and one PCO dimax S4. The frame rate of each camera is between 200 and 1000 Hz, depending on the experiment. This paper presents only experiments with a frame rate of 600 Hz. A summary of the camera properties is shown in Table 1.

The same traverse as the PIV system is used as a mounting system (see Figure 1). The cameras are positioned outside the wind tunnel outlet to avoid interaction with the flow. This restriction results in camera baselines of 3.5 m. Four LED lamps (IES 4438 LED Sync Lamp) from IES Elektronikentwicklung are used to illuminate the field of view (2 m × 2 m). Each lamp is pulsed through the high-speed cameras and illuminates the scene with 2.2 klx for 50 µs. The illumination time is equal to the exposure time of 50 µs, which reduces motion blur effects significantly.

A microcontroller module is used to synchronize the cameras. The module provides a TTL signal for each camera image with a specific frame rate for synchronous image exposure. A so-called Synchronometer with a temporal resolution of 10 µs, as shown in [60], is used to check the synchronization quality. Simulations on the given setup with an assumed asynchrony of 10 µs result in an accuracy of 0.4 mm in object space. In several evaluations, no asynchrony could be determined between cameras, resulting in a synchronization accuracy of fewer than 10 µs.

Artificial targeting of the blades is necessary because their natural patterns are very homogeneous. In this study, two different types of targeting are used. One target type is made by white dots, which signal discrete points. The benefits are a high precision center estimation and low weight. Retro-reflective targets can be used for optimal reflection quality. However, these have a height of 120 µm and are therefore unsuitable for small-scale aerodynamic applications. Instead, we use targets made of matte foil with a height of 80 µm and a diameter of 15 mm. A total of 12 targets per blade are used, which have a distance of 25 mm to 83 mm from each other. A lightweight solution is essential in these applications because the additional weight directly impacts the behavior of the blades. The dot targeting increased the weight of the blades by 6% (5 g in total). A speckle pattern is used as the second type of targeting to reconstruct the whole surface. In this way, the blade deformations can be derived with a high spatial resolution. However, the foil leads to changed blade properties. Firstly, the weight is increased by 10 g. Secondly, the stiffness of the blades changes due to the coating. Therefore, the experiments with dot signalization are presented in this article.

**Figure 4:**
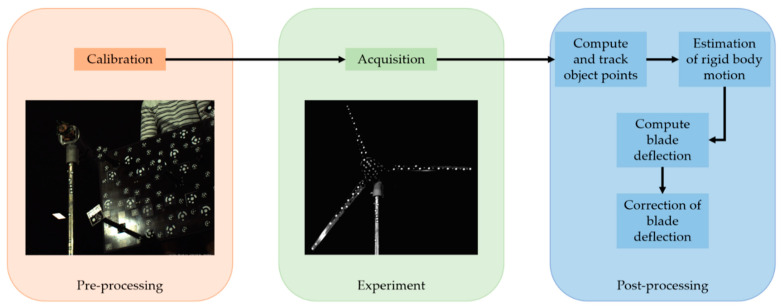
Processing chain of photogrammetry.

Figure 4 shows the processing of the photogrammetry data. The first step consists of the camera calibration. A self-calibration bundle adjustment is used to determine the interior and exterior orientations. The model of bundle adjustment is extended by the constraints of a fixed relative orientation [61]. The commercial software MoveInspect Pilot from AICON 3D Systems processes the acquired image sequences. The object coordinates are based on a spatial forward intersection and are tracked with an LSM algorithm. Due to temporary occlusions and the high similarity of the measurement targets, the tracking leads to erroneous trajectories. Therefore, a new tracking method is used, described in Section 4.3. In addition to the corrected trajectories, the method is used to determine the rigid-body motions of the wind turbine. These motions (tower motion and self-rotation) affect the complete system, leading to an incorrect deformation analysis. The rigid body motions are corrected through a 3D similarity transformation from the respective epochs. The trajectories of the individual points now contain the three-dimensional motion resulting from aerodynamic effects. These are reduced by the reference positions of the respective measurement targets to obtain the deformations. The reference positions result from the photogrammetric acquisition of a static scene which is characterized by zero wind loads on the turbine.

### 3.4. Characteristic of FSI-System

The photogrammetry and PIV systems represent the whole measurement system for acquiring FSI. The most important properties are summarized in Table 2, where the values are heavily dependent on the camera and measurement setup.

The Nyquist theorem is used to calculate the temporal resolution. The temporal resolution results from half of the respective frame rate of the used camera system. To calculate the resolution of the spatial fluid behavior, for example eddies, the size of the interrogation area is multiplied by the ground sample distance (GSD). The interrogation area has a size of 32 pixels × 32 pixels, which results in a geometric resolution of 2 mm. A deformation of the object leads to a different position on the image sensor. Therefore, the geometric resolution λ of deformation depends on the image measurement accuracy σ and the GSD. Equation (1) shows this relationship, whereby the introduced factor is based on GUM [62].

(1) λ=σ·3·GSD

This factor provides that the change of the image point is a deformation and not a measurement uncertainty. The image measurement accuracy can be assumed to be 5/100 pixels for circular measurement targets, resulting in a resolution of 0.15 mm [61]. However, it should be noted that this is a theoretical value. In practice, the detection of significant deformations is primarily limited by the accuracy of the measurement system, especially the calibration accuracy of interior and relative orientation. Notwithstanding the image measurement, the accuracy depends on the camera configuration and the quality of the calibration process. It is assumed to be 0.5 mm for this highly dynamic application.

## 4. Challenges and Methods

Although the aspects of synchronization and the determination of the coordinate system play a minor role in the acquisition of static surfaces, these are essential tasks in the acquisition of dynamic surfaces. The complexity of these questions increases with the acquisition of FSI. Thus, a general time reference and a global coordinate system are required for a holistic observation. For this purpose, the two measurement systems must be combined both in time and in space.

### 4.1. Synchronization

**Figure 5:**
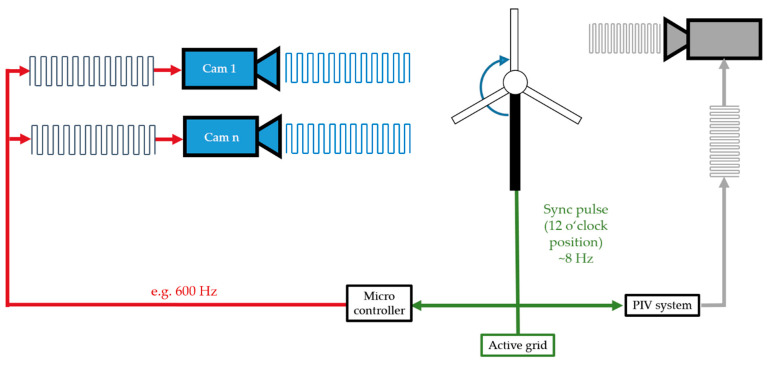
Scheme of synchronization of the experimental setup.

For the analysis of the FSI, synchronization of all measurement systems, namely the active grid and MoWiTO is necessary. The concept of synchronization is illustrated in Figure 5. The protocol of the active grid defines the start trigger (t0). At the start of the experiment, the active grid sends a TTL signal to the measurement systems and the MoWiTO. Due to the configuration of the light sheet of the PIV system, flow data can only be acquired at the 12 o’clock position. Therefore, the data acquisition does not start directly at the t0 signal with the predefined acquisition rate. Instead, both measurement systems start with data acquisition at the first passing of the 12 o’clock position of a rotor blade. Both systems can be used with a different frame rates.

The described concept was experimentally validated by using a synchronometer. This device generates high-frequency light patterns for high-speed camera images. It was shown that the asynchrony between the measurement systems is less than 1 µs. However, it should be noted that the model wind turbine does not rotate at a constant speed due to the dynamic inflow. The inconsistent rotational speed can lead to a drift effect, which results in the flow data recordings not being stationary. Therefore, the MoWiTO trigger resets the timestamps of the measurement systems at each rotation to achieve synchrony at the 12 o’clock blade position. Due to the new initialization, the new trigger can arrive at the cameras before completing the last acquisition. This behavior leads to deviations from the defined photogrammetric frame rate between the last and first image of subsequent rotations. The theoretical maximum deviation is the reciprocal of the frame rate for each rotation and depends on the rotation speed of the MoWiTO. The inconstant frequency also rules out the use of a phase-locked loop (PLL) and requires this specific solution to the inconsistent data acquisition rates. Determination and correction of these deviations are done in post-processing by evaluating the timestamps of the image sequence. The resolution of the timestamps is 1 µs, which means that the frame rate can be assumed to be sufficiently constant. The exact 12 o’clock blade position can be interpolated with the stored trigger time series.

### 4.2. Coordinate Systems

**Figure 6:**
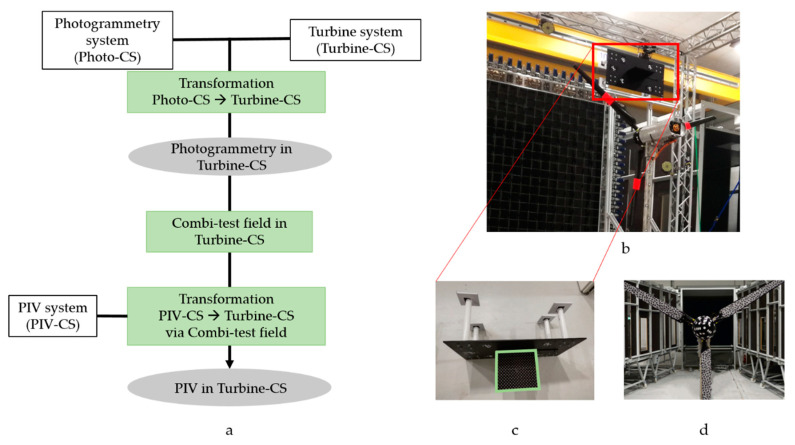
Transformation procedure (**a**); Combi-test field in operation (**b**); Combi-test field, PIV calibration field in green (**c**); Nacelle with targets (**d**).

In order to be able to combine the results of the two measurement systems in the space domain, a global coordinate system is required. The global coordinate system is called Turbine-CS and has its origin at the tip of the nacelle. The alignment of the coordinate axes is based on the DNV-GL guideline and is fixed to the tower direction [63]. Here, the X-axis corresponds to the axis of rotation, and the Z-axis faces in the tower’s direction. The Y-axis completes the left-handed coordinate system. For the realization of the coordinate system, the nacelle is assumed to be a rigid body marked by measurement targets (Figure 6d). A photogrammetric bundle adjustment carries out the determination of the coordinates. These are required to determine the six parameters of a similarity transformation of Photo-CS and PIV-CS coordinate systems.

Figure 6a shows the procedure for estimating the transformation parameters. Photo-CS describes the local coordinate system of the photogrammetry system. The origin and orientation of the coordinate system are the same as the exterior orientation of one camera. The calculation of the transformation parameters is based on the points on the nacelle. These points are given in Photo-CS and Turbine-CS. The PIV-CS describes the local coordinate system of the PIV system and is defined by a calibration field (see Figure 6c). However, determining the parameters for the transformation into Turbine-CS is not directly possible. For example, the measurement targets are outside the FOV of the photogrammetry system due to the perpendicular camera configuration. In addition, the depth of field is only a few millimeters large and is aligned with the laser plane. A new test field (Combi-test field) is used to determine the transformation parameters. The Combi-test field consists of the PIV calibration field (green frame) extended by photogrammetric measurement targets (see Figure 6b,c). The PIV calibration field’s object coordinates and measurement targets are determined beforehand by evaluating a closed-loop configuration in the PIV-CS. The targets of the Combi-test field can be determined simultaneously by the photogrammetry system during the PIV system’s calibration process. Afterward, the PIV system can be transformed into the Turbine-CS because the photogrammetry system is already known in the Turbine-CS. All measurement systems are thus available in one global coordinate system.

### 4.3. Estimation of Rigid-Body Motion

**Figure 7:**
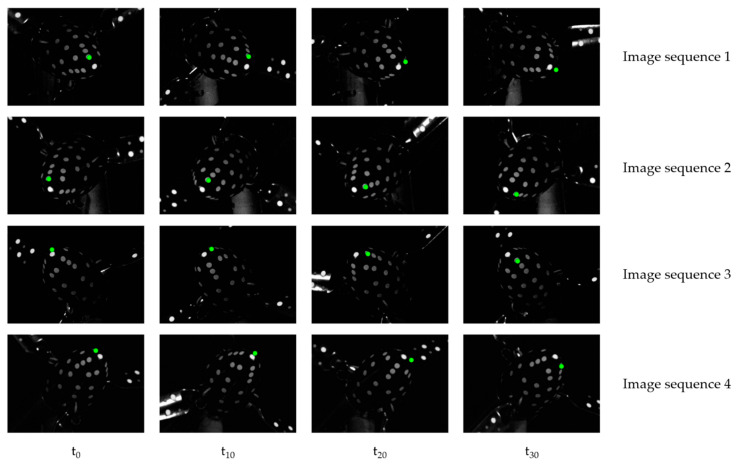
Image sequence: the green point is tracked through four epochs, showing complicated tracking behavior due to occlusions and similarity between uncoded targets.

Another challenge in acquiring FSI on rotating wind turbines is the photogrammetric deformation analysis. As already described in Section 3.3, the signaling influence on the blades should be kept to a minimum. Therefore, circular targets are used for the signalization of the turbine. Furthermore, the influence of the tower motion and self-rotation must be eliminated for the deformation analysis. However, the measurement targets on the flexible rotor blades cannot be used to determine the rigid body motions. Only the wind turbine’s nacelle can be identified as a rigid body affected by the same rigid body effects as the blades. The evaluation showed that tracking the measurement targets leads to erroneous trajectories due to the high similarity and temporary occlusions. An image sequence of the measurement targets on the nacelle is shown in Figure 7 to illustrate this effect.

Although the green target is visible in the images of the first and second camera in the first epoch t_0_, it is hidden in the images of cameras three and four. Due to its rotation, the target position changes in the subsequent epochs (t_10_–t_30_). The target “walks” from the field of view of images 1 and 2 into images 3 and 4. Furthermore, the figure shows that the targets are close to each other due to the limited object size, which favors mismatching. Therefore, a new tracking method is used that is robust against temporary occlusions and closely spaced targets.

The algorithm is organized in several steps, as seen in Pseudo code 1. The algorithm uses the object coordinates Xp_t_ of the targets in the different epochs as input data. In addition, the object coordinates of the nacelle points Xp_ref_ from the reference epoch are needed. The method’s goal is to predict the points Xp_ref_ in each epoch t. Using a nearest-neighbor search algorithm, each predicted nacelle point Xp_pred_ is attempted to be matched with a point Xp_t_. The matching takes place in image space. The matching in the image domain can reduce the list of potential matching partners by reducing the dimensions and a visibility check. Furthermore, robustness can be increased by using epipolar constraints and spatio-temporal loop closures. The rigid body motions are determined if three or more points can be matched. For this purpose, a RANSAC-based six parameter transformation is used [64]. All object coordinates of the rotor blade points are transformed by inverting the determined transformation parameters (RANSAC-6DOF). A nearest-neighbor search algorithm performs the matching to the reference epoch in object space.

**Pseudo code 1.** Estimation of rigid body motion.**Input** xp_ref,... #Reference coordinates xp_t,... #Object coordinates in epoch t image_sequence,... campara_list #Interior and exterior orientation ekf_global=init_global_ekf() global_storage=[] #List of rigid body motion xp_tracks=[] #Transformed object coordinates   **For each t**∈**image_sequence**     
xp_pred=ekf_global.Predict(xp_ref,t)    
list=[]     **For each i**
∈
**xp_pred**    
#Find corresponding points on nacelle       
list(i)=Find_nearest_neighbor2d(xp_pred(i),... xp_t(t),campara_list, image_sequence(t))     **end**     **If Size(list)**
≥
**3**    
#Compute and save rigid body motion       
x=RANSAC_6DOF(list)      
ekf_global.Update(list)      
global_storage(t)=x       
#Transform object coordinates       
xp_blade=Transformation(x, xp_t(t))       **For each i **
∈
**xp_ref**      
#Find corresponding object coordinates of each blade       
xp_blade(i)=Find_nearest_neighbor(xp_ref(i),...      
xp_blade)       **end**       
xp_tracks(t)=xp_blade     **end**   **end** **Output **
global_storage, xp_tracks

The basis for the method is the prediction in the object domain. The prediction of the next time step is notating with t+1. Different models can be used for the prediction. Models with linear assumptions (e.g., constant rotational speed) are unsuitable for this application. Instead, an Extended Kalman Filter (EKF) is used, an established method for pose estimation [65,66]. EKF assumes a rigid-body motion to describe the motion of all measurement targets on the nacelle. The state vector $\vec{x}$ contains 12 parameters (3 translations, 3 rotations, and 6 velocities).

(2) $$\vec{x}={\left[x,y,z,\omega ,\varphi ,\kappa ,x_{v},y_{v},z_{v},\omega_{v},\varphi_{v},\kappa_{v}\right]}^{T}$$

The prediction is made by the transition matrix T, which models the velocity of the state vector.

(3) $$\vec{x_{t+1}}=T\vec{x}\cdot +\cdot C\vec{w}$$

(4) $$S_{\bar{xx},t+1}=TS_{x}T^{T}+CS_{ww}C^{T}$$

with

(5) $$T=\left[\begin{array}{cc} I & I\cdot t\\ 0 & I \end{array}\right]$$

The tuning of the filter is done by the correctly corresponding points of Xp_ref_ and Xp_t_. The filter update is implemented according to the known scheme after [67].

The filter design results in several advantages for the tracking. First, only one filter is needed to predict all nacelle points. Points that cannot be measured over a more extended period can thus still be confidently predicted at any epoch. Furthermore, single incorrect matches do not corrupt the filter behavior. Another advantage is the possibility of integrating further rigid body information. For example, measured rotation angles can be directly integrated and thus lead to a shorter initial duration and an increase in the robustness of the filter.

## 5. Results

The measurement campaigns aimed to realize the experimental setup and the described concept in real wind tunnel experiments. More than 80 data sets were acquired during two measurement campaigns, corresponding to a data volume of about 20 TB. Furthermore, data sets could be recorded in different flow situations. In the context of this publication, the recorded information is shown but not analyzed in more detail concerning aerodynamic effects. The obtained inflow and deformation information can form the basis for future investigations of FSI and other aerodynamic effects. The analysis of the aerodynamic effect will be the focus of future publications.

The first measurement campaign is characterized by the simultaneous use of the PIV system and the photogrammetry system. In the second campaign, only the photogrammetry system was available. The results are comparable since both measurement campaigns could realize the same experiment conditions (e.g., inflow properties and temperature). The settings of the measurement systems are identical for all experiments, according to Section 2. Furthermore, strain gauge measurements could be performed in both campaigns for the individual blades, which measure the global forces of each blade.

In general, accuracy validation of a dynamic measurement system is complex due to missing reference systems or bodies with high accuracy. Additionally, the multi-step processing procedure limited the statistical accuracy validation. Therefore, the validation is based on known inflow conditions, whose interaction with the wind turbine is known.

### 5.1. Flow Measurement

**Figure 8:**
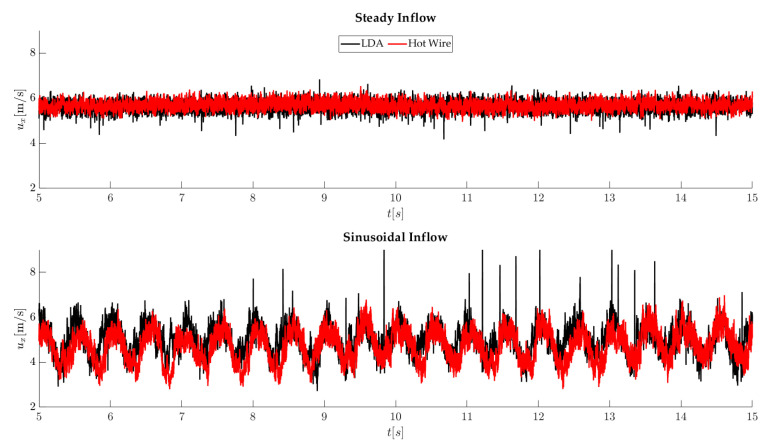
Wind flow profiles.

Hot wire and LDA measurements are used to measure the inflow. Two of the used flow types are shown in Figure 8. Both flow types have a duration of 30 s. A constant velocity of 5.6 m/s, a turbulence intensity of 3%, and an integral length scale of 25 cm characterize a steady wind flow. The fluctuations are based primarily on the active grid. This inflow results in a relatively constant load on the rotor blades, where a constant deflection can be expected. The second inflow is called sinusoidal. The characteristic is a sinusoidal behavior with an amplitude of 1 m/s and frequency of 0.5 Hz. This inflow results in an alternating load on the rotor blades, which should also correspond to a sinusoidal profile.

**Figure 9:**
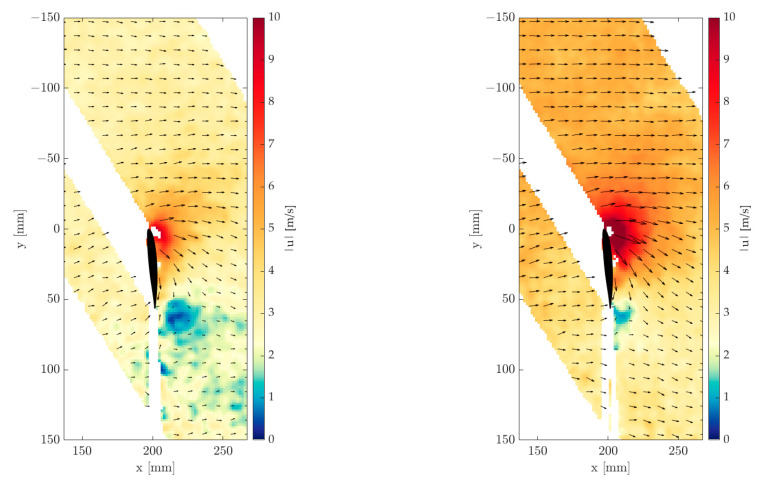
Processed flow field in sinusoidal inflow. Minimum of sinusoidal inflow (**left**) and maximum of sinusoidal inflow velocity (**right**).

As an example, Figure 9 shows processed flow fields measured during sinusoidal inflow. The velocities are colored, and the flow directions are illustrated with arrows. Since the blade crosses the light sheet, a shadow is cast. These shadowed areas cannot be evaluated and are empty in the figures. The flow fields are acquired at the same blade position but during different states of the sinusoidal inflow. Thus, the left image shows a flow field at the minimum of the flow modulation. Here, two areas are conspicuous in which the wind speed and direction deviate from the inflow. Significantly higher wind speeds of up to 8 m/s can be detected on the rotor blade, some of which extend over the entire blade surface. In contrast, in the bottom right area of the image, areas can be observed where very low wind speeds and no dominant wind direction exist.

Figure 9 right shows a flow field at a maximum wind speed of the sinusoidal inflow. In contrast to the left figure, low wind speeds only occur at one location. Furthermore, the high velocities at the rotor blade are significantly higher and reach a maximum of up to 10 m/s.

### 5.2. Deformation

**Figure 10:**
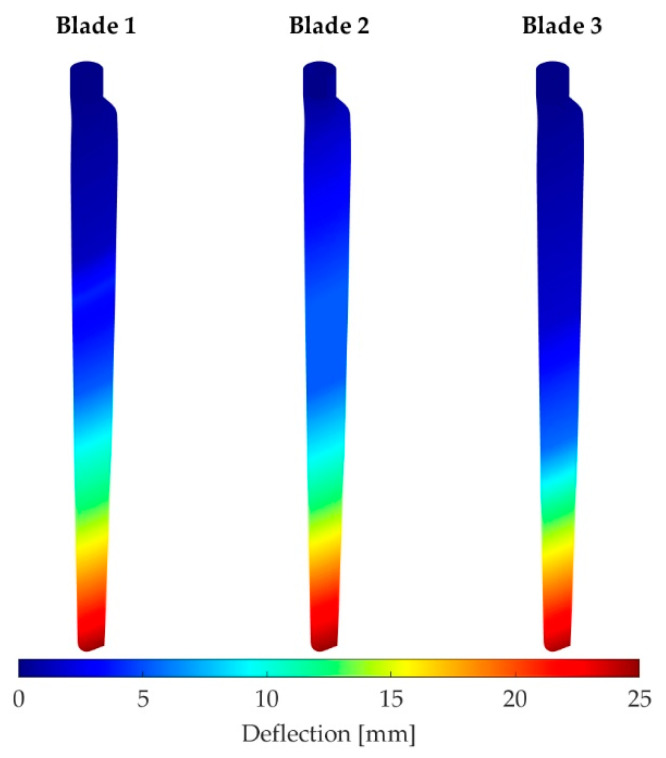
Global blade deflection with steady inflow.

In the following, the deformations of the rotor blades in steady and sinusoidal flow are shown as examples. Figure 10 shows the deformation for the whole blade surfaces of all three blades in steady wind conditions. The deflections are obtained by interpolating the motion of the discrete points, which are disturbed over the entire surface. Due to the different positions of the targets on each blade, the deformation section varies a little bit. However, all three blades show a similar behavior during the experiments. Furthermore, the deformation section shows a slightly diagonal profile based on the perspective view of the blades. In general, no deflection can be seen on the root. The deflection increased with the distance to the root, so the highest deflections are on tip positions. This behavior can be seen in all data sets. In addition, the standard deviation of the deflection is similar in all datasets, with results up to 0.3 mm. However, based on the camera configuration, the redundancy is low, and the standard deviation may be unreliable.

For a better overview, only the deformations of the tip position in the wind direction (X-axis) are shown in the following figures. These have the most significant motions due to the stiffness model of the blades.

#### 5.2.1. Steady Inflow

**Figure 11:**
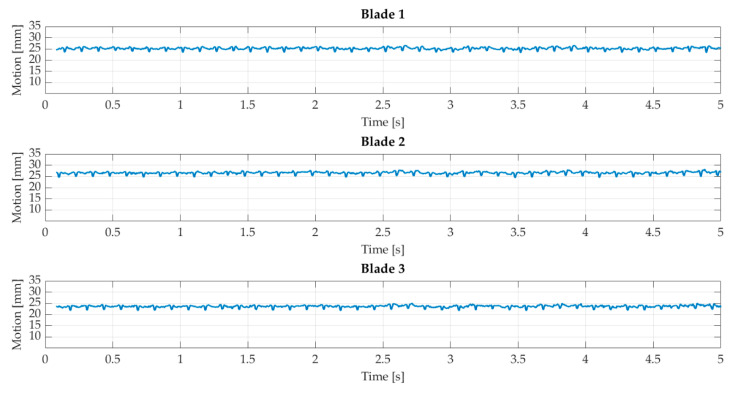
Blade deflection with steady inflow.

**Figure 12:**
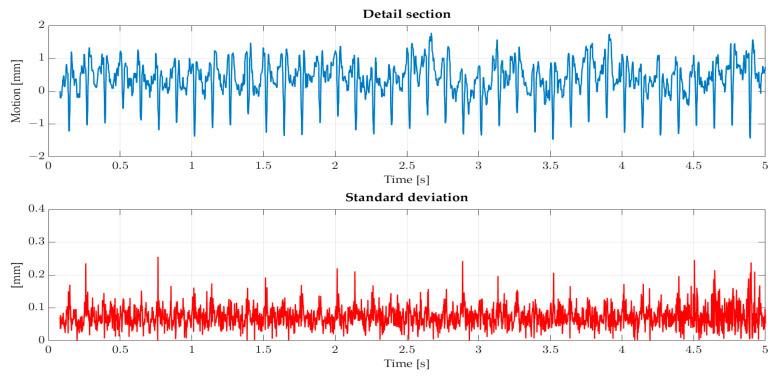
Detail of section of blade deflection with steady inflow without the constant offset is shown as a blue profile. The standard deviation is shown as a red profile. Both profiles have different scaling.

Figure 11 shows the resulting deformation with steady inflow. All three blades show similar behavior and a constant offset. The offset is 25 mm and is caused by the average loads on the blades, which based on the inflow and rotation of the turbine. The deflection profile is linear, which is expected by steady inflow. The LED illumination needs approximately 0.08 s to reach full power in each experiment. This initialization process results in dark images and a gap of 0.08 s of data. Furthermore, alternating deformation can be seen in the profiles, which are visible in the detail section in Figure 12.

The deformations are up to 2 mm and have a frequency of approximately 8 Hz. This behavior can be explained by the passing of the tower. During the passing, the aerodynamic behavior changes, which impacts the deflection. In addition, the standard deviation of the tip point in the wind direction (X-axis) is shown. The standard deviation of the two other components is smaller by factor 2. The standard deviation profile is almost linear and means that the blade’s behavior can be determined with the same precision at every blade position. Compared to the motion, the standard deviation is slight, with a maximum of 0.25 mm and a mean value of 0.072 mm. As mentioned, redundancy is low and does not allow a comprehensive accuracy analysis. However, the expected behavior of the blades in steady inflow and minor aerodynamic effects can be determined.

#### 5.2.2. Sinusoidal Inflow

**Figure 13:**
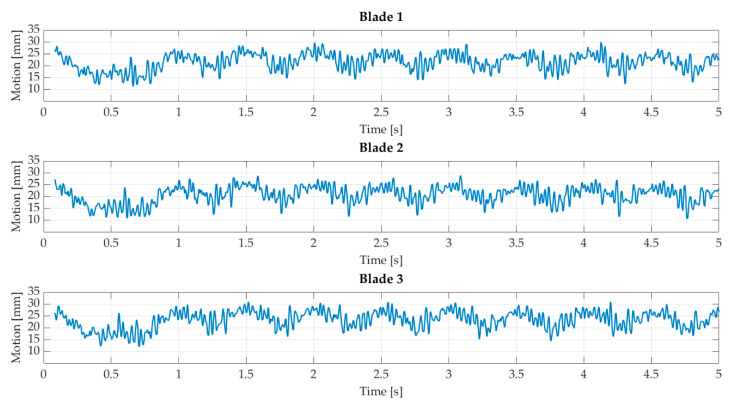
Blade deflection with sinusoidal inflow.

Figure 13 shows the deformation profile with sinusoidal inflow. The expected characteristic deformation behavior can be seen for all three blades. The maximum deflection is 31 mm, and the constant deflection is approximately 20 mm. In comparison to the steady flow, the constant deflection is smaller. However, the tower effect can also be seen in this setup. In addition, the constant deflection of the three blades varies. This effect can probably be explained by different target positions on the respective blades and a minor misalignment of the turbine.

**Figure 14:**
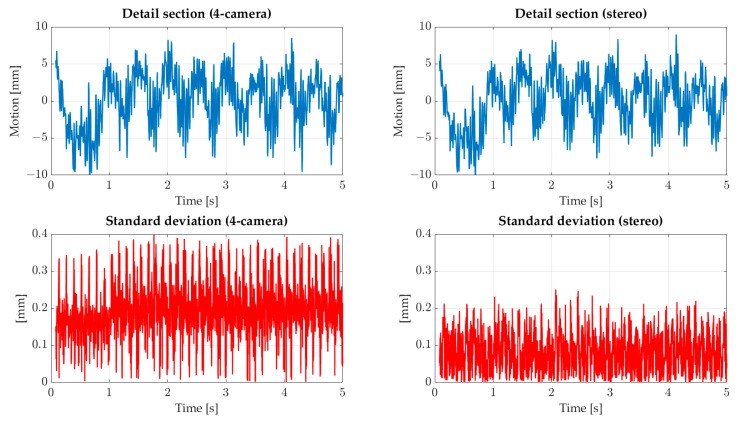
Detail section of motion profile by sinusoidal inflow is shown as a blue profile. The standard deviation is shown as a red profile and is balanced by the mean value. Motion and standard deviation have different scaling.

In Figure 14, a detailed section and the standard deviation profile can be seen. The detail section is balanced with the mean value without the constant offset. The motions lie in an interval from −10 mm to 5 mm, where the minimum is reached within the first second. After the first second, the profile shows a sinusoidal profile and a minimum of −5 mm.

Compared to the steady experiment, the standard deviation of the whole camera system (left) is conspicuous. The standard values are much higher and reach a maximum of 0.4 mm. The mean value is 0.19 mm, almost a factor of 3 higher than the steady case. However, the number of cameras used in each step is 3 or 4, comparable to the steady case. In addition, high and low peaks can be seen at the 6 o’clock position. These peaks can be explained by the low quality of the image measurement. Furthermore, the positions of the points are near the image borders of the cameras, which increases the impact of distortion. If only the upper cameras are used as a stereo system, the determined movements are comparable to the complete camera system (see Figure 14 detail section (stereo)). The standard deviations for the stereo system are in a similar range as for the steady inflow with an average of 0.077 mm. Therefore, the lower cameras are probably responsible for the increased standard deviations. However, it is necessary to analyze the camera calibrations and their influence on the complete processing pipeline.

#### 5.2.3. Comparison PIV and Photogrammetry

The deformation is determined by image correlation using PIV for a data set with sinusoidal inflow for further comparison. The blade deformation can only be determined at the 12 o’clock position for the PIV system. Although the deformations are determined for 10 s with the PIV system, the photogrammetry system could only acquire data for 5 s.

**Figure 15:**
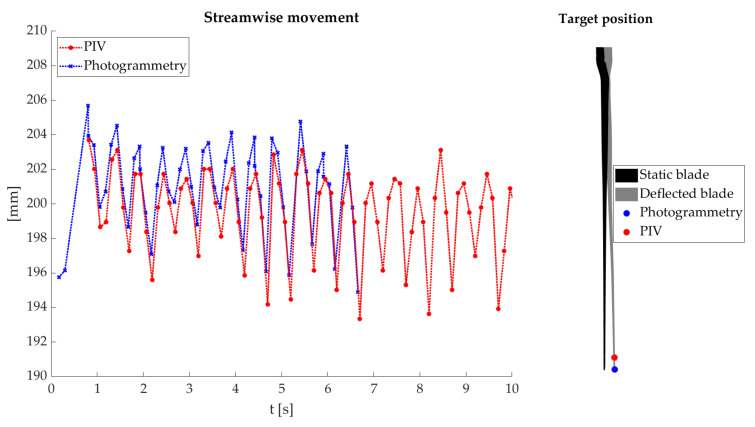
Deflection comparison with PIV system (**left**). Different target positions lead to a different X-axis position (**right**).

The deformations are shown in Figure 15. Comparing the two profiles indicates a high degree of similarity and an expected sinusoidal profile. However, a slightly systematic deviation of 2 mm can be seen. The deviations can probably be explained by different measurement positions and uncertainties in the transformation process. The impact of different measurement positions can be split into two parts. The first part results in a different perspective of both measurement systems for the blade. The PIV system observes the blade surface at the suction side. The photogrammetry system measures at the pressure side, meaning that the thickness of the blade leads to a systematic deviation. Figure 15 (right) illustrates the second part from different measurement positions. The measurement targets (blue) are used for the deformation analysis through the photogrammetry system, which can be determined precisely. Instead, the PIV system uses a manually selected point (red) at the light sheet on the other blade surface. The photogrammetry target cannot be located at the same position caused by the red protection foil. Therefore, both measurement points are not on the same deformation line. The deformation line of the rotor blades takes an exponential curve. The different positions mean that even minor deviations along the blade length can lead to systematic deviations.

Another aspect is the uncertainty of the transformation process. These uncertainties of the transformation process affect the combination of both measurement methods. The translation’s uncertainty systematically affects the combination at each position in the measurement volume. Instead, uncertainties of the rotation have a leverage effect and affect the tip positions primarily. The influence of the leverage effect depends on the position of the Combi-test field. In this case, the Combi-test field is located near the nacelle, and uncertainties of 0.03° lead to an error of 0.5 mm at the tip position. Therefore, a highly accurate transformation is needed to combine both techniques. However, the comparison and previous results show a high degree of similarity and the success of the concept.

## 6. Discussion and Conclusions

Fluid-structure interactions play a significant role in many applications. However, acquiring the phenomenon in real environments and validating different models is complex. This paper presents a setup to record FSI on a rotating wind turbine model in wind tunnel experiments. This approach is characterized by the simultaneous acquisition of fluid flows and blade deformations in a global coordinate system. The experimental setup consists of two optical 3D measurement methods. A stereo PIV system (2D-3C) is used to detect the fluid flow. A photogrammetric system is used to acquire the blade deformations. Different challenges are addressed, e.g., synchronization, realizing a global coordinate system, and the photogrammetric deformation analysis. The blade deformations interfere with the rigid body motions of the model turbine. Therefore, a new processing approach has been developed to separate the rigid body motion and deformation from each other. This approach aims to determine the rigid body motions in a two-step procedure and correct the blade motions afterward. The approach is robust against occlusions and allows the integration of further kinematic information (e.g., rotational velocities). However, it should be noted that the rigid body motions must be determined with high accuracy to identify fine-scale deformations. Translation deviations can systematically change the deformations. On the other hand, inaccurate rotation angles lead to leverage effects, particularly corrupt deformations at the blade tips. In order to show the effects of minor uncertainties in the process chain on the resulting blade deformations, a statistical analysis is necessary. However, this task is highly complex due to the dynamic characteristics of the application.

The realization of the experimental setup could be shown through different experiments. These experiments demonstrated that the blade deformations correspond to the expectations and reach high statistical accuracy. However, the standard deviation is higher than expected for sinusoidal inflow. The standard deviation can probably be explained by the different image measurement quality and the influence of lens distortion. Further statistical and numerical analysis is needed to evaluate the impact of measurement and calibration quality on the complete processing pipeline. For a first numerical comparison, the deflection of the blades is estimated with both systems. The comparison showed a high degree of similarity. However, a systematic deviation can be seen, which may be caused by different target positions and uncertainties in the transformation process. Nevertheless, the experiments prove the concept and the ability to acquire information of FSI with high accuracy and high temporally and spatially resolution.

In addition, data sets with turbulent inflow are acquired. In this way, aerodynamic and geometric information for steady, sinusoidal, Mexican-hat, and turbulent inflow are available for the investigation of FSI. The data can also be used for comparison with simulation results. Comparisons of this type can help to improve engineering models and simulation codes which will increase the efficiency of these methods. Furthermore, the presented measurement system allows the holistic view of other aerodynamic effects of wind turbines, which cannot be recorded in field experiments or only with very high effort.

An extension of the PIV system to a 3D-3C system is theoretically possible. It would provide further important information for a better understanding of wind turbines. However, several technical challenges must be addressed, and new evaluation algorithms must be developed.

## Figures and Tables

**Table 1:** Camera properties.

Camera	Sensor Size	Lens	Exposure Time
PCO Dimax HD+	1920 Pixel × 1440 Pixel 21.12 mm × 15.84 mm	35 mm Zeiss	50 µs
PCO Dimax S4	2016 Pixel × 2016 Pixel 22.18 mm × 22.18 mm	35 mm Zeiss	50 µs

**Table 2:** Properties of the measurement setup.

**PIV-System**	**Temporal resolution of wind flow**	**FOV**	**Pixel size in object domain (GSD)**	**Geometric resolution of flow behavior**
	2000 Hz	150 mm × 300 mm	0.057 mm	2 mm
**Photogrammetry**	**Temporal resolution of deflection**	**FOV**	**Pixel size in object domain (GSD)**	**Geometric resolution of deflection**
	300 Hz	2000 mm × 2000 mm	1 mm	0.15 mm (0.5 mm)

## References

[1] Borowski F., Sämann M., Pfensig S., Wüstenhagen C., Ott R., Kaule S., Siewart S., Grabow N., Schmitz K.-P., Stiehm M. (2018). Fluid-structure interaction of heart valve dynamics in comparison to finite-element analysis. Curr. Dir. Biomed. Eng..

[2] Winzen A., Roidl B., Schröder W. (2015). Particle-image velocimetry investigation of the fluid-structure interaction mechanisms of a natural owl wing. Bioinspiration Biomim..

[3] Roul R., Kumar A. (2020). Fluid-Structure Interaction of Wind Turbine Blade Using Four Different Materials: Numerical Investigation. Symmetry.

[4] Zhang L., Sun C. (2018). Simulation Analysis of Fluid-Structure Interaction of High Velocity Environment Influence on Aircraft Wing Materials under Different Mach Numbers. Sensors.

[5] Zhang P., Huang S. (2011). Review of aeroelasticity for wind turbine: Current status, research focus and future perspectives. Front. Energy.

[6] Hsu M.C., Bazilevs Y. (2012). Fluid–structure interaction modeling of wind turbines: Simulating the full machine. Comput. Mech..

[7] Mydlarski L. (2017). A turbulent quarter century of active grids: From Makita (1991) to the present. Fluid Dyn. Res..

[8] Eloranta H., Pärssinen T., Saareninne P. (2005). Fluid–structure interaction of a splitter plate in a convergent channel. Exp. Fluids.

[9] Hoerner S., Bonamy C. (2019). Structured-light-based surface measuring for application in fluid-structure interaction. Exp. Fluids.

[10] Huynh D., McKeon B. (2020). Measurements of a turbulent boundary layer-compliant surface system in response to targeted, dynamic roughness forcing. Exp. Fluids.

[11] Nila A., Phillips N., Bomphrey R.J., Bleischwitz R. Optical Measurements of Fluid-Structure Interactions for Description of Nature-Inspired Wind Dynamics. Proceedings of the RAeS Applied Aerodynamics Conference.

[12] Nietiedt S., Göring M., Willemsen T., Wester T., Kröger L., Gülker G., Luhmann T. (2019). Measurement of fluid-structure interaction of wind turbines in wind tunnel experiments: Concept and first results. ISPRS Int. Arch. Photogramm. Remote Sens. Spat. Inf. Sci..

[13] Premalatha M., Abbasi T., Abbasi S.A. (2014). Wind energy: Increasing deployment, rising environmental concerns. Renew. Sustain. Energy Rev..

[14] Hansen M.O.L., Sørensen J.N., Voutsinas S., Sørensen N., Madsen H.A. (2006). State of the art in wind turbine aerodynamics and aeroelasticity. Prog. Aerosp. Sci..

[15] Snel H., Schepers J.G., Montgomerie B. (2007). The MEXICO project (Model Experiments in Controlled Conditions): The database and first results of data processing and interpretation. J. Phys. Conf. Ser..

[16] Carrión M., Woodgate M., Stejil R., Barakos G., Gómez-Iradi S., Munduate X. (2014). CFD and Aeroelastic Analysis of the MEXICO Wind Turbine. J. Phys. Conf. Ser..

[17] Plaza B., Bardera R., Visiedo S. (2015). Comparison of BEM and CFD results for MEXICO rotor aerodynamics. J. Wind Eng. Ind. Aerodyn..

[18] Wester T.T.B., Kampers G., Gülker G., Peinke J., Cordes U., Tropea C., Hölling M. (2018). High speed PIV measurements of an adaptive camber airfoil under highly gusty inflow conditions. J. Phys. Conf. Ser..

[19] Ragni D., van Oudheusden B.W., Scarano F. (2012). 3D pressure imaging of an aircraft propeller blade-tip flow by phase-locked stereoscopic PIV. Exp. Fluids.

[20] Cardano D., Carlino G., Cogotti A., Schröder A., Willert C.E. (2008). PIV in the Car Industry: State-of-the-Art and Future Perspectives. Particle Image Velocimetry: New Developments and Recent Applications.

[21] Hoskins P.R. (1990). Measurement of arterial blood flow Doppler ultrasound. Clin. Phys. Physiol. Meas..

[22] Battisti L., Zanne L., Dell’Anna S., Dossena V., Persico G., Paradiso B. (2011). Aerodynmaic Measurements on a Vertical Axis Wind Turbine in a Large Scale Wind Tunnel. J. Energy Resour. Technol..

[23] Bayati I., Bernini L., Zanotti A., Belloli M., Zasso A. (2018). Experimental investigation of the unsteady aerodynamics of FOWT through PIV and hotwire wake measurements. J. Phys. Conf. Ser..

[24] Raffel M., Willert C.E., Scarano F., Kähler C.J., Wereley S.T., Kompenhans J. (2018). Particle Image Velocimetry: A Practical Guide.

[25] Zhang Z. (2002). Velocity bias in LDA measurements and its dependence on the flow turbulence. Flow Meas. Instrum..

[26] Kitzhofer J., Ergin F.G., Jaunet V. 2D Least Squares Matching applied to PIV Challenge data (Part 1). Proceedings of the 16th International Symposium on Applications of Laser Techniques to Fluid Mechanics.

[27] Willneff J. (2003). A Spatio-Temporal Matching Algorithm for 3D Particle Tracking Velocimetry. Ph.D. Thesis.

[28] Maas H.-G. (1992). Digitale Photogrammetrie in Der Dreidimensionalen Strömungsmesstechnik. Ph.D. Thesis.

[29] Cai S., Liang J., Zhou S., Gao Q., Xu C., Wei R., Wereley S., Kwon J. Deep-PIV: A new framework of PIV using deep learning techniques. Proceedings of the 13th International Symposium on Particle Image Velocimetry—ISPIV 2019.

[30] Kröger L., Wester T.T.B., Langidis A., Nietiedt S., Göring M., Luhmann T., Peinke J., Hölling M., Gülker G. (2020). Experimental study of fluid-structure interaction at a model wind turbine blade using optical measurement techniques. J. Phys. Conf. Ser..

[31] Taylor Z.J., Gurka R., Kopp G.A., Liberzon A. (2010). Long-Duration Time-Resolved PIV to Study Unsteady Aerodynamics. IEEE Trans. Instrum. Meas..

[32] Brücker C. (1997). 3D scanning PIV applied to an air flow in a motored engine using digital high-speed video. Meas. Sci. Technol..

[33] Kähler C.J., Kompenhans J. (2000). Fundamentals of multiple plane stereo particle image velocimetry. Exp. Fluids.

[34] Sacrano F. (2012). Tomographic PIV: Principles and practice. Meas. Sci. Technol..

[35] Elsinga G.E., Sacrano F., Wieneke B., Van Oudheusden B.W. (2006). Tomographic partile image velocimetry. Exp. Fluids.

[36] Maas H.G., Stefanidis A., Gruen A. (1994). From pixels to voxels: Tracking volume elements in sequences of 3D digital images. Int. Arch. Photogramm. Remote Sens..

[37] Westfeld P., Maas H.G., Pust O., Kitzhofer J., Brücker C. 3-D Least Squares Matching for Volumetric Velocimetry Data Processing. Proceedings of the 15th International Symposium on Applications of Laser Techniques to Fluid Mechanics.

[38] Scarano F., Ghaemi S., Giuseppe C.A.C., Bosbach J., Dierksheide U., Sciacchitano A. (2015). On the use of helium-filled soap bubbles for large-scale tomographic PIV in wind tunnel experiments. Exp. Fluids.

[39] Shortis M.R., Robson S., Jones T.W., Goad W.K., Lunsford C.B. (2016). Photogrammetric tracking of aerodynamic surfaces and aerospace models at NASA Langley Research Center. ISPRS Ann. Photogramm. Remote Sens. Spat. Inf. Sci..

[40] Liebold F., Maas H.-G. (2017). Sub-pixel accuracy crack width determination on concrete beams in load tests by triangle mesh geometry analysis. ISPRS Ann. Photogramm. Remote Sens. Spat. Inf. Sci..

[41] Bethmann F., Luhmann T. (2017). Object-based Semi-global Multi-image Matching. PFG J. Photogramm. Remote Sens. Geoinf. Sci..

[42] Baltsavias E.P. (1991). Multiphoto Geometrically Constrained Matching. Ph.D. Thesis.

[43] Schwalbe E. (2013). Entwicklung von Verfahren zur Bestimmung räumlich-zeitlich hochaufgelöster Bewegungsvektorfelder an Gletschern aus monoskopischen Bildsequenzen. Ph.D. Thesis.

[44] D’ Apuzzo N. (2003). Surface Measurement and Tracking of Human Body Parts from Multi Station Video Sequences. Ph.D. Thesis.

[45] Bolles R., Woodfill J. (1993). Spatio-temporal consistency checking of passive range data. Int. Symp. Robot. Res..

[46] Borer D., Delbruck T., Rösgen T. (2017). Three-dimensional particle tracking velocimetry using dynamic vision sensors. Exp. Fluids.

[47] Vo M., Narasimhan S.G., Sheikh Y. Spatio-temporal bundle adjustment for dynamic 3d reconstruction. Proceedings of the IEEE Conference on Computer Vision and Pattern Recognition.

[48] Hung C.H., Xu L., Jia J. (2013). Consistent binocular depth and scene flow with chained temporal profiles. Int. J. Comput. Vis..

[49] Dong Y.L., Pan B. (2017). A Review of Speckle Pattern Fabrication and Assessment for Digital Image Correlation. Exp. Mech..

[50] Pan B., Xie H., Wang W., Qian K., Wang Z. (2008). Study on subset size selection in digital image correlation for speckle patterns. Opt. Express.

[51] Lecompte D., Smits A., Bossuyt S., Sol H., Vantomme J., Van Hemelrijck D., Habraken A.M. (2006). Quality assessment of speckle patterns for digital image correlation. Opt. Lasers Eng..

[52] Salvi J., Fernandez S., Pribanic T., Lladi X. (2010). A state of the art in structured light patterns for surface profilometry. Pattern Recognit..

[53] Zhang S. (2018). High-speed 3D shape measurement with structured light methods: A review. Opt. Lasers Eng..

[54] Li B., Zhang S. (2017). Superfast high-resolution absolute 3D recovery of a stabilized flapping flight process. Opt. Express.

[55] Li H., Waldman R.M., Zhang K., Hu H. Quantification of Dynamic Water Droplet Impact onto a Solid Surface by using a Digital Image Projection Technique. Proceedings of the 55th AIAA Aerospace Sciences Meeting.

[56] Kröger L., Frederik J., Van Wingerden J.W., Peinke J., Hölling M. (2018). Generation of user defined turbulent inflow conditions by an active grid for validation experiments. J. Phys. Conf. Ser..

[57] Neuhaus L., Hölling M., Bos J.T.W., Peinke J. (2020). Generation of Atmospheric Turbulence with Unprecedentedly Large Reynolds Number in a Wind Tunnel. Phys. Rev. Lett..

[58] Berger F., Kröger L., Onnen D., Petrović V., Kühn M. (2018). Scaled Wind Turbine Setup in a Turbulent Wind Tunnel. J. Phys. Conf. Ser..

[59] Langidis A., Nietiedt S., Berger F., Kröger L., Petrović V., Wester T.T.B., Gülker G., Göring M., Rofallski R., Luhmann T. (2022). Design and evaluation of rotor blades for fluid structure interaction studies in wind tunnel conditions. J. Phys. Conf. Ser..

[60] Raguse K. (2007). Dreidimensionale photogrammetrische Auswertung asynchrony aufgenommener Bildsequenzen mittels Punktverfolgungsverfahren. Ph.D. Thesis.

[61] Luhmann T., Robson S., Kyle S., Boehm J. (2020). Close-Range Photogrammetry and 3D Imaging.

[62] Joint Committee for Guides in Metrology (2008). Evaluation of Measurement Data—Guide to the Expression of Uncertainty in Measurement.

[63] (2015). Rotor Blades for Wind Turbines.

[64] Fischler M.A., Bolles R.C. (1981). Random Sample Consensus: A Paradigm for Model Fitting with Applications to Image Analysis and Automated Cartography. Comm. ACM..

[65] Nützi G., Weiss S., Scaramuzza D., Siegwart R. (2011). Fusion of IMU and vision for absolute scale estimation in monocular SLAM. J. Intell. Robot. Syst..

[66] Alatise M.B., Hancke G.P. (2017). Pose Estimation of a Mobile Robot Based on Fusion of IMU Data and Vision Data Using an Extended Kalman Filter. Sensors.

[67] Heunecke O., Kuhlmann H., Welsch W., Eichhorn A., Neuner H., Möser M., Müller. G., Schlemmer. H (2013). Auswertung geodätischer Überwachungsmessungen. Handbuch Ingenieurgeodäsie.

